# Dynamic visualization of membrane-inserted fraction of pHluorin-tagged channels using repetitive acidification technique

**DOI:** 10.1186/1471-2202-10-141

**Published:** 2009-11-30

**Authors:** Serguei S Khiroug, Evgeny Pryazhnikov, Sarah K Coleman, Andreas Jeromin, Kari Keinänen, Leonard Khiroug

**Affiliations:** 1 Neuroscience Center, University of Helsinki, FIN-00014, Helsinki, Finland; 2 Department of Biological and Environmental Sciences, Division of Biochemistry, University of Helsinki, FIN-00014, Helsinki, Finland; 3 Allen Institute for Brain Science, Seattle, WA 98103, USA

## Abstract

**Background:**

Changes in neuronal excitability, synaptic efficacy and generally in cell signaling often result from insertion of key molecules into plasma membrane (PM). Many of the techniques used for monitoring PM insertion lack either spatial or temporal resolution.

**Results:**

We improved the imaging method based on time-lapse total internal reflection fluorescence (TIRF) microscopy and pHluorin tagging by supplementing it with a repetitive extracellular acidification protocol. We illustrate the applicability of this method by showing that brief activation of NMDA receptors ("chemical LTP") in cultured hippocampal neurons induced a persistent PM insertion of glutamate receptors containing the pHluorin-tagged GluR-A(flip) subunits.

**Conclusion:**

The repetitive acidification technique provides a more accurate way of monitoring the PM-inserted fraction of fluorescently tagged molecules and offers a good temporal and spatial resolution.

## Background

The ability to monitor changes in the amount of key proteins residing in PM is crucial for understanding neuronal function and synaptic plasticity, but existing methods have several restrictions. Total internal reflection fluorescence (TIRF) microscopy has been employed to monitor protein trafficking to and from PM [1-4]. The TIRF method uses the so called evanescent field to excite fluorescence within 100-200 nm above the glass bottom of a culture dish. However, the visibility of a fluorescent molecule in a TIRF image does not necessarily mean that the molecule is inserted in the PM, because many intracellular organelles located near the PM are well within the evanescent field. Indeed, TIRF microscopy readily visualizes unfused secretory vesicles [5], lysosomes [6], mitochondria [7,8] and the endoplasmic reticulum (ER) [9,10].

Another technique for monitoring PM insertion of fluorescent molecules is based on pHluorin tagging [11-13]. Fluorescence of ecliptic pHluorin, the multiple-point mutant of EGFP, is completely quenched at pH below 6.0 [14]. The key assumption of this technique is that pHluorin is fully quenched while in the lumen of secretory organelles [15], whereas upon PM insertion the tagged molecules pHluorin regains fluorescence due to exposure to extracellular milieu (pH_o_~7.4). In practice, this assumption is incorrect because the lumen of many intracellular organelles, notably the ER, is not acidic but has pH around 7.2 [16]. Hence, pHluorin-tagged molecules located in the ER exhibit bright fluorescence, which may add a strong background and thus "contaminate" the fluorescent signal of the PM-inserted molecules. Since fluorescence of intracellular and extracellular pHluorin-tagged molecules often overlaps, the imaging results are prone to misinterpretation.

Two groups have recently employed TIRF imaging to monitor PM insertion of pHluorin-tagged AMPA receptors [17,18]. While offering a greatly improved sensitivity, this assay is not, in principle, devoid of image contamination caused by the unquenched pHluorin residing in non-acidic intracellular compartments. In the present study, we solved this problem by using repetitive acidification tests in combination with TIRF imaging and pHluorin tagging of GluR-A-containing AMPA receptors.

## Results and Discussion

**Figure 1:**
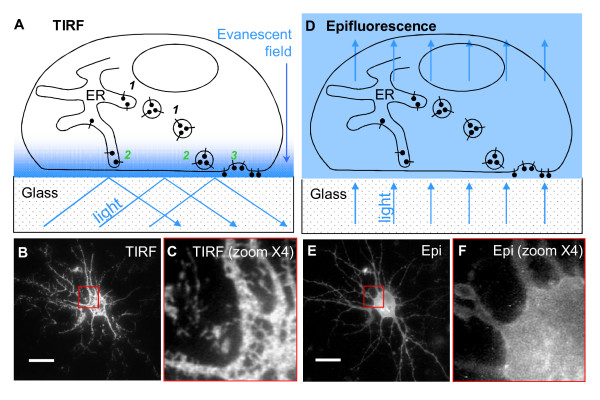
**TIRF imaging highlights the perimembrane fraction of pHluorin-tagged GluR-A receptors**. **A**, schematic representation of TIRF imaging of pHluorin-tagged molecules located either in the endoplasmic reticulum (ER) and vesicles or inserted in the PM. The pHluorin-tagged molecules that are close enough to the PM to be excited by evanescent field are marked with numbers 2 (ER-residing molecules) and 3 (membrane-inserted molecules and those residing in the perimembrane vesicles). The more distant pHluorin-tagged molecules that are not visualized in TIRF imaging mode are marked with number 1. **B**, example of TIRF image of a neuron transfected with pHluorin-GluR-A revealing individual clusters (magnified field is shown in **C**). **D**, schematic representation of epifluorescence imaging of pHluorin-tagged receptors located either in the endoplsasmic reticulum (ER) and vesicles or inserted in the PM. Note that all pHluorin-tagged receptors irrespective of their subcellular localization are visualized in epifluorescence mode. **E**, epifluorescence image of the same neuron that appears in B (magnified field of the epifluorescence image is shown in **F**). Note that the spatial resolution of individual clusters in epifluorescence is inferior compared to TIRF imaging mode (B and C). Scale bars: 10 μm.

We transfected cultured hippocampal neurons with the glutamate receptor subunits GluR-A(flip) tagged with pHluorin on the extracellular N-terminus (pHluorin-GluR-A; Additional file 1: Figure S1). A similar construct has been employed in a recent study, except the authors used the *flop *splice variant [17]. In order to highlight the membrane-inserted fraction of these receptors, we combined total internal reflection fluorescence (TIRF) microscopy with pHluorin tagging as has been done previously [17,18]. The TIRF microscopy selectively visualizes fluorescent molecules located within approximately 0.1-0.2 μm of the cell-bearing glass coverslip (therefore, in or close to the basal PM; Figure 1A-C). Unlike epifluorescence imaging where all fluorescent molecules in a cultured cell are excited by the light passing through the cell (Figure 1D-F), TIRF offers supra-optical vertical resolution superior to that of confocal microscopy [19]. Indeed, individual clusters of pHluorin-GluR-A fluorescence were clearly visible in TIRF (Figure 1C) but not in epifluorescence mode (Figure 1F).

While in some secretory organelles the fluorescence of pHluorin molecules is fully quenched by acidic intralumenal milieu [14,15], quenching of the ER-residing pHluorin may not occur because pH in the ER is not acidic [16]. To test directly whether the ER-residing pHluorin retains its fluorescence, we overexpressed in cultured hippocampal neurons a pHluorin-encoding construct engineered for expression in the lumen of endoplasmic reticulum (pHluorin-ER). This protein is retained in the ER via retrograde transport based on KDEL receptor (see Methods). We observed bright fluorescence of the pHluorin-containing ER (Additional file 2: Figure S2). Importantly, pHluorin-ER was detectable not only in epifluorescence but also in TIRF mode (Additional file 2: Figure S2), consistent with our previous findings [5,7]. This observation lead us to conlcude that i) fluorescence of the ER-resident pHluorin molecules is not quenched, and that ii) TIRF imaging mode does not fully exclude the fluorescence of intracellular pHluorin-tagged molecules.

**Figure 2:**
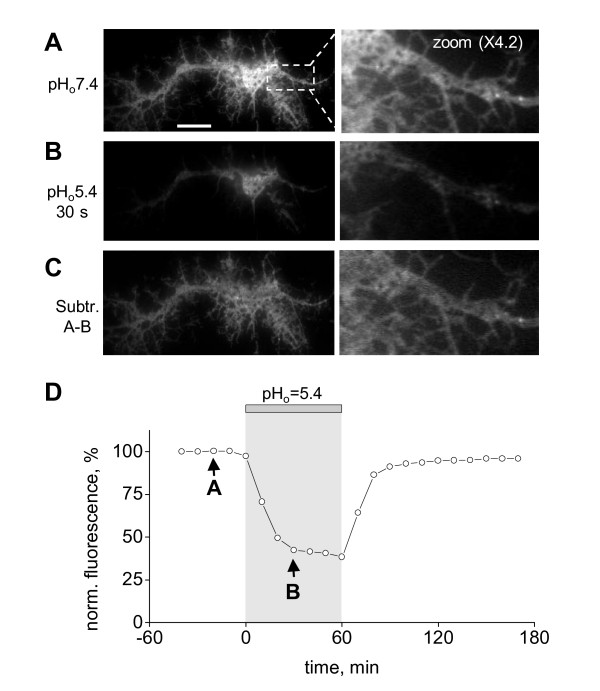
**Manipulations of extracellular pH reveal distinct fractions of pHluorin-tagged GluR-A receptors visualized in TIRF mode**. Right panel shows zoomed images of the transfected neuron presented in the left panel. **A**, pHluorin-GluR-A clusters under control conditions (pH_o _= 7.4). Scale bar: 5 μm. **B**, image obtained 30 seconds into the application of acidic solution (pH_o _= 5.4) shows strong but incomplete quenching of the pHluorin-GluR-A fluorescence. **C**, calculated image obtained by subtracting B from A. **D**, plot of changes in fluorescence intensity (averaged across the whole imaging field). Time points referring to each of the images shown in A and B are indicated by arrows with corresponding letters.

To overcome these limitations, we set out to develop a method for assessing the fractional contribution of the PM-inserted pHluorin-GluR-A receptors *versus *intracellular ones to the overall fluorescence. We performed repetitive acidification tests, which consisted in perfusing cells for 1-2 minutes with an acidic extracellular solution (pH_o _= 5.4, titrated with HCl). The overall pHluorin fluorescence was strongly reduced within 30 seconds of the acidification test (compare Figure 2A and 2B). The rate of fluorescence quenching closely corresponded to the time required for near-complete exchange of the bath solution, which was determined in a separate set of experiments (22 ± 3 s, n = 3; see Methods). This observation strongly suggests that the rapid drop in fluorescence was caused by quenching of the PM-inserted pHluorin-GluR-A molecules due to their direct exposure to extracellular acidic solution.

In spite of the rapid initial quenching, a significant fraction of the pHluorin-GluR-A receptors retained their fluorescence during the acidification tests (Figure 2B). On average, in 39 neurons the fluorescence fraction that remained unquenched after 30 seconds of the acidification test constituted 42 ± 3%. The unquenched fluorescence was observed throughout the neuronal soma and neurites (Figure 2B) and often had a reticular appearance resembling that of the ER. To visualize spatial distribution of those pHluorin-GluR-A molecules that were quenched during the acidification test, we digitally subtracted the image obtained during the test (Figure 2B) from the control image (Figure 2A). The resulting image (Figure 2C) showed fluorescence that was distributed throughout neuronal soma and dendritic tree, a pattern consistent with the PM-inserted fraction of pHluorin-GluR-A [18].

When the acidification test was terminated by returning to the control perfusion solution (pH_o _= 7.4), the fluorescence level was completely restored to its pre-test value within few minutes (time course of a representative experiment is illustrated in Figure 2D). Repetitive 1- or 2-minute acidification tests did not have any irreversible effects on the overall pHluorin fluorescence, because complete fluorescence recovery was reliably observed when we repeated these tests for up to one hour with the 5 minute inter-test intervals (data not shown). It is conceivable that repetitive acid exposure could have caused some subtle changes in those physiological parameters of neurons that were not monitored in our experiments. However, reproducibility of acidification tests argues against accumulating detrimental effects.

**Figure 3:**
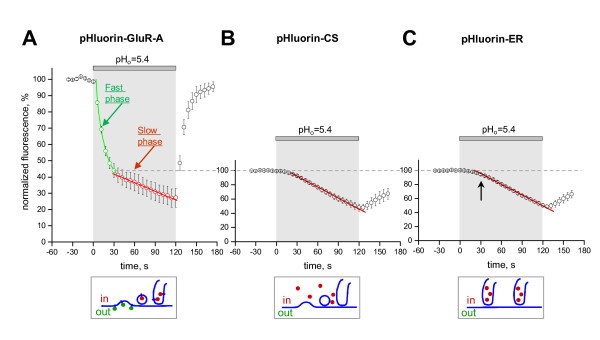
**Biphasic changes in pHluorin fluorescence induced by application of acidic solution (pH_o _= 5.4)**. **A**, trace showing averaged time course of the pHluorin-GluR-A fluorescence quenching (normalized to the fluorescent intensity at the pre-application level). The initial, fast phase of fluorescence quenching is marked by green circles, the subsequent slow phase by red circles. Green and red lines represent best fitting curves for fast phase (exponential decay) and slow phase (linear regression), respectively. **B**, averaged time course of the cytosolic pHluorin (pHluorin-CS) quenching induced by application of acidic solution. The values are normalized to the fluorescence intensity at the pre-application level. Red line represents best linear fit for the plot. **C**, averaged time course of quenching of the pHluorin trapped in endoplasmic reticulum (pHluorin-ER) induced by application of acidic solution. Values are normalized to the fluorescence intensity at the pre-application level. Red line represents best linear fit for the plot. Note that the kinetics are similar both to these of pHluorin-CS (B) and to the slow phase of pHluorin-GluR-A fluorescence quenching (red circles in A). Schematic representation of the cellular localization of pHluorin-tagged molecules for each of the construct is shown at the bottom of each panel.

Next, we focused on kinetic analysis of the pHluorin-GluR-A fluorescence changes during the acidification test. We hypothesized that this analysis may provide a means for repetitive measurement of the PM-inserted fraction of pHluorin-GluR-A receptors. Using a shorter inter-frame interval (6 s), we performed acidification tests and recorded the time course of the fluorescence quenching and its recovery (Figure 3A). The initial decrease in the fluorescence was termed "fast phase" (Figure 3A, green circles) because it developed rapidly with a mono-exponential decay time constant of 13.4 ± 0.8 s (R^2 ^= 0.999; n = 3). Thus, by 30 seconds after the beginning of the test, the fast phase was largely over (i.e., ~90% completed). After the first phase, we observed the second phase of fluorescence quenching that proceeded more gradually (Figure 3A, red circles). We termed this second phase "slow phase" because its time course was slower than that of the fast phase (decay time-constant of 69.2 ± 3.7 s, n = 3; P < 0.0001). While the exponential equation was a satisfactory fit for the slow phase (R^2 ^= 0.989), the linear fit was more suitable (R^2 ^= 0.995) and yielded the linear coefficient of 0.17 ± 0.03%/s (n = 3).

Fractional contributions of the fast and slow phases varied widely between different cells and different subcellular regions. We hypothesized that larger fractional contributions by the slow phase were due to stronger "contamination" of the "signal" (i.e., fluorescence of the PM-inserted pHluorin-GluR-A receptors) by "noise" (i.e., fluorescence of the receptors residing in non-acidic organelles such as the ER). If this is true, then the slow phase of fluorescence quenching (red circles in Figure 3A) should reflect passive acidification of these organelles, which is secondary to the decrease in cytosolic pH caused, in turn, by extracellular acidification. To test this hypothesis, we transfected neurons with a construct encoding for soluble pHluorin expressed in the cytosol (pHluorin-CS). A two-minute perfusion with acidic solution (pH_o _= 5.4) produced a slow, gradually developing decrease in pHluorin-CS fluorescence (Figure 3B). This decrease was well fitted with a linear regression (R^2 ^= 0.994), with the coefficient of 0.47%/s. This result indicates that, at a later stage of the pH test, the partial collapse of the proton gradient across the PM caused gradual acidification of the cytosol.

The cytosolic acidification should then lead to acidification of those intracellular organelles that lack active proton transport. We transfected cells with the ER-trapped pHluorin (pHluorin-ER), performed acidification tests and compared the time course of fluorescence changes in neurons expressing pHluorin-ER (Figure 3C) to those expressing pHluorin-CS (Figure 3B) or pHluorin-GluR-A (red circles in Figure 3A). Similarly to pHluorin-CS, the pHluorin-ER fluorescence exhibited a slow decrease during the acidification test and was well fitted with a linear regression (R2 = 0.991), yielding the linear coefficient of 0.45%/s. Importantly, the near-linear time course of the fluorescence changes both in the cytosol and ER was very similar to the slow phase of pHluorin-GluR-A fluorescence quenching (red circles in Figure 3A). Indeed, the linear coefficients were close for all three constructs. To compare them, one should take into account the fractional contribution of the pHluorin-GluR-A slow phase that was, on average, 42% (Figure 3A). Thus, the corrected linear coefficient for the slow phase of pHluorin-GluR-A quenching is 0.17%/s/0.42 = 0.40%/s, which corresponds well to the linear coefficients of both pHluorin-CS (0.47%/s) and pHluorin-ER (0.45%/s). Taken together, these results strongly suggest that the slow phase of pHluorin-GluR-A fluorescence quenching was due to intracellular acidification, and that this slow phase developed after the fast phase was largely complete.

The clear temporal segregation of fast and slow quenching phases allowed us to perform accurate separation of the fluorescent signal derived from the PM-inserted pHluorin-GluR-A receptors from that of the intracellular receptors. For our repetitive acidification method, we selected the time point of 30 seconds after the beginning of the acidification test because at this point approximately 90% of the fast quenching phase is completed, whereas the contribution of the slow phase is negligible. We estimated the error due to the slow phase contribution to be below 3%. This estimate is based on two measurements: i) thirty seconds after the beginning of the acidification test, the decrease in pHluorin-ER fluorescence (black arrow in Figure 3C) was 5.8 ± 1.1% (n = 5), and ii) on average, the intracellular fraction of the pHluorin-GluR-A fluorescence was 42%. Thus, the error of our method can be calculated as 5.8% × 0.42 = 2.4%. Importantly, the acidification tests are highly reproducible and thus can be used for monitoring changes in the PM-inserted fraction of pHluorin-tagged channels, such as their insertion or internalization during synaptic plasticity.

To demonstrate applicability of the repetitive acidification method, we used it to monitor translocation of pHluorin-GluR-A receptors to the PM. Membrane insertion of GluR-A-containing AMPA receptors associated with LTP is a well-established phenomenon (for review see [20]). In a recent study, Yudowski and colleagues used pHluorin tagging to visualize membrane insertion of GluR-A(flop) receptors [17]. Here, we focused on the "flip" splice variant of this receptor. On hippocampal neurons transfected with pHluorin-GluR-A, we performed repetitive acidification tests with a 10 minute interval. During each test, we measured the amplitude of the fast quenching phase 30 seconds after the onset of acidification.

**Figure 4:**
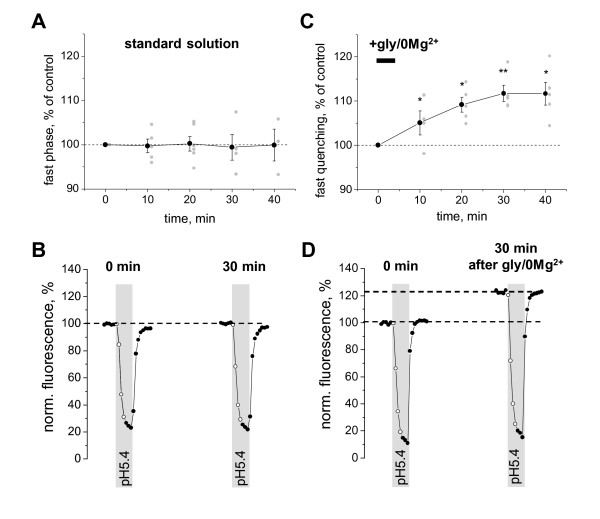
**Effects of glycine-mediated NMDA receptor activation on PM insertion of pHluorin-GluR-A**. **A**, plot of normalized amplitude of the fast quenching phase during the pH test. The plot shows averaged data (black circles with error bars) overlaid with individual data points (grey circles) from 6 cells. No statistically significant differences between time points were observed (P > 0.05), indicating that the PM-inserted receptor fraction was stable over at least 30 minutes under control conditions. **B**, a pair of representative traces of pHluorin-GluR-A fluorescence obtained with a 30 min interval under control conditions. **C**, averaged data plot (n = 8 cells) showing glycine-induced long-lasting changes in the fast quenching phase amplitude. Thirty minutes after the start of a brief (5 minute; horizontal bar) application of the glycine/0 Mg^2+ ^solution, the fast phase amplitude significantly increased compared to non-treated cells (P < 0.01) indicating persistent insertion of additional pHluorin-GluR-A receptors in the PM. **D**, typical traces showing glycine-induced increase in the fast phase of fluorescence quenching, as well as a rise in the baseline fluorescence. Both effects are consistent with an increase in the PM-inserted fraction of pHluorin-GluR-A receptors.

Under control conditions, the amplitude of the fast phase remained stable for at least 40 minutes (Figure 4A). We stimulated NMDA receptors by removing extracellular Mg^2+ ^and adding glycine (200 μM) for 5 minutes. After this stimulation commonly used to trigger the so-called "chemical LTP" [17,21], we observed a long-lasting increase in the fast phase amplitude (Figure 4C). Thirty minutes after the glycine/0 Mg^2+ ^application, fast quenching phase was significantly larger compared to control conditions (after glycine: 112 ± 2%, n = 5; control: 99 ± 3%, n = 4; P < 0.01). Significant difference was maintained at 40 minutes (P < 0.05). At some subcellular locations we observed much larger increases in the fast phase amplitude (up to 180%; data not shown). However, the detailed analysis of region specificity of GluR-A membrane insertion was beyond the scope of this study. Taken together, these results demonstrate persistent insertion of pHluorin-GluR-A(flip) receptors to the PM in response to brief activation of NMDA receptors. They also show that the repetitive acidification method can be used as a tool to monitor kinetics of activity-dependent AMPA receptor membrane trafficking.

**Figure 5:**
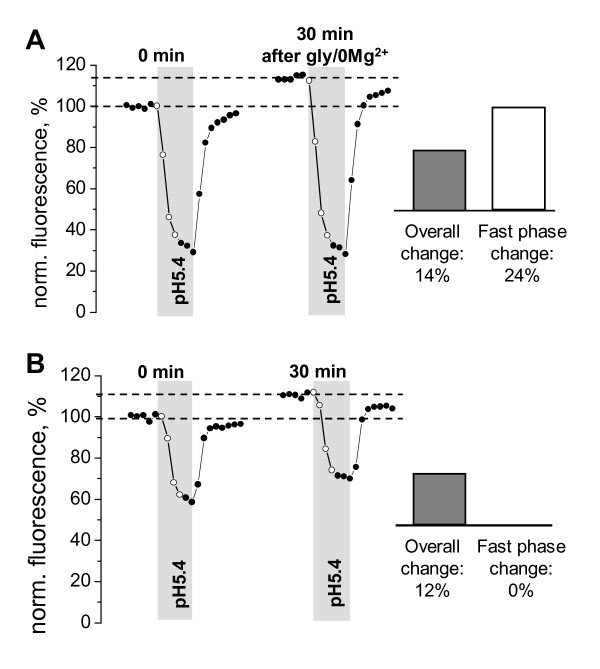
**Repetitive pH tests allow separation of true changes in the PM-inserted fraction of pHluorin-GluR-A receptors from insertion-unrelated fluctuations in the overall fluorescence**. **A**, an example of glycine-induced changes in pHluorin-GluR-A fluorescence, which demonstrates that monitoring of changes in overall fluorescence may lead to underestimation of the actual increase in PM-inserted receptor fraction. In contrast, monitoring changes in the fast phase of fluorescence quenching allows a more accurate estimate of true changes in the PM-inserted fraction. The respective values are shown as bars in the right panel. **B**, an example from another neuron that was monitored under control conditions (i.e., without applying gly/0 Mg^2+^) showing that an increase in the overall fluorescence is not always indicative of the PM insertion of new receptors. The bars in the right panel demonstrate that the amplitude of the fast phase of fluorescence quenching remains unchanged in spite of an increase in baseline fluorescence.

Two observations presented below illustrate advantages of our method over the previously used approach where changes in the overall pHluorin-GluR-A fluorescence were used as the index of membrane insertion [17,18]. First, we found that repetitive acidification method has an improved sensitivity due to the increase in the signal-to-noise ratio. This is apparent in the example shown in Figure 5A. Thirty minutes after gly/0 Mg^2+ ^application, insertion of additional pHluorin-GluR-A receptors to the PM resulted in i) increased overall fluorescence as well as ii) increased amplitude of fast quenching phase. The increase in the overall fluorescence constituted 14%: fluorescence level grew from 100 to 114 on the normalized fluorescence scale, (114-100)/100 = 14%. The increase in the fast phase amplitude was considerably larger and constituted 24%, calculated as: first test 100-38 = 62, second test 114-37 = 77, change (77-62)/62 = 24%. The reduced sensitivity of the conventional method is most likely caused by the "contamination" of the overall fluorescence by intracellular pHluorin-GluR-A receptors. In contrast, the fast phase amplitude only reflects membrane-inserted pHluorin-GluR-A molecules, thus making the sensitivity of the repetitive acidification method superior. An alternative way to remove the background fluorescence that is contributed by unquenched intracellular pHluorin consists in pre-bleaching and has been previously employed by von Zastrow's group [17]. In this approach, prolonged light exposure is used to destroy fluorophore of the unquenched pHluorin-GluR-A molecules (PM-inserted and intracellular alike) while sparing the fluorophore of the quenched pHluorin-GluR-A molecules and preserving their ability to become fluorescent upon PM insertion [17].

The second notable advantage of the repetitive acidification method is that it allows distinguishing between PM-insertion-related changes in pHluorin fluorescence from those caused by e.g. changes in the pH of the intracellular organelles containing pHluorin-tagged molecules. In the example of Figure 5B, the neuron was monitored under control conditions without NMDA receptor activation. During the 30 minutes between the first and the second acidification tests (left and right traces, respectively) the overall pHluorin-GluR-A fluorescence increased by (112-100)/100 = 12%. In contrast, the amplitude of the fast quenching phase remained unchanged: first test 100-62 = 38, second test 112-74 = 38, change (38-38)/38 = 0%. This observation suggests that interpreting the increase in pHluorin fluorescence as an indication of membrane insertion may lead to erroneous conclusions and should, therefore, always be verified.

## Conclusion

The use of repetitive acidification tests allows avoiding several pitfalls of the existing methods. In a number of recent studies, the increase in the brightness of the TIRF image has been attributed to insertion of additional fluorescent molecules in the PM [1,4]; here, we demonstrated that the PM-associated TIRF image can be strongly "contaminated" by the background fluorescence of molecules located in non-acidic organelles, such as the ER. The mere visibility of pHluorin-tagged molecules has often been interpreted as evidence of their PM residence [11,13]; we now showed that as many as half of the pHluorin molecules visible in TIRF images of cultured hippocampal neurons may actually reside in intracellular compartments with near-neutral pH (Figure 2B and Figure 3A). It is also worth noticing that the fast quenching phase amplitude was measured as percentage of the overall fluorescence, i.e. as a ratio. Like any ratiometric technique, this method allows direct comparison between different regions of the same cell or between cells expressing different constructs. Although we did not pursue detailed spatial analysis in this study, future research will greatly benefit from the repetitive acidification method because it provides information on the extent of PM insertion at different subcellular sites.

## Methods

### 2.1 DNA constructs

Standard molecular biological techniques, including PCR-assisted mutagenesis were used to make expression plasmids. Expression plasmids for ER-retained pHluorin and N-terminally pHluorin-tagged rat GluR-A were constructed in pcDNA3.1(-) (Stratagene). Briefly, pHluorin cDNA was inserted between a signal peptide derived from rat GluR-D (residues 1-21; P19493) and cDNA encoding the mature polypeptide of GluR-A (residues 19-907; flip isoform; P19490). To make pHluorin-ER, an ER-lumen resident form of pHluorin, KDEL tetrapeptide sequence was added to the carboxyterminus of pHluorin encoded by GluR-D signal peptide - pHluorin expression cassette. The cytosolic form of pHluorin, pHluorin-CS, with no signal peptide or any other sequence modifications was constructed in pcDNA3.1. All constructs were verified by restriction mapping and by sequencing through all PCR-derived parts.

### 2.2 Preparation and transfection of rat hippocampal neurons

All experimental procedures were approved by the Animal Care and Use Committee, University of Helsinki. Cultured neurons were prepared from embryonic day 18 rat hippocampi. Hippocampi were dissociated with Papain solution (10 U/mL). The cells were plated at a density of 3 × 10^4 ^cells cm^2 ^on glass-bottomed Petri dishes (MatTek) pre-coated with poly-L-lysine and laminin (1-2 μg/cm^2^). Cultures were maintained in the 5% CO_2_/95% air atmosphere at 37°C in Neurobasal medium (Invitrogen; pH = 7.4) supplemented with B27 (Invitrogen), 0.5 mM L-glutamine, 100 units/mL penicilline and 100 μg/mL streptomycine. Medium was changed twice per week. Neurons were transfected after 6-10 days *in vitro *with constructs using Lipofectamine 2000 (Invitrogen) according to the manufacturer's instructions. Lipofectamine 2000 was removed after 6-8 hours. Cells were analyzed 3-5 days after transfection.

### 2.3 Fluorescence imaging

For TIRF imaging experiments, cell-containing MatTek dishes were transferred to the CellR imaging system (Olympus Europe, Hamburg, Germany). The system was equipped with an automated filter wheel for excitation filters and with a 488 nm (20 mW) DPSS laser (Melles Griot, CA, USA) for TIRF imaging. The microscope frame and all optical elements were maintained at 34°C using the temperature control incubator (Solent Scientific, Segensworth, UK). Images were collected with a CCD camera (Orca, Hamamatsu, Japan). In TIRF mode fluorescence was excited by the thin evanescent field formed above the glass substrate due to total internal reflection of the laser beam (attenuated to 5-10%). Frames were acquired every 3 to 10 seconds.

### 2.4 Drug application and acidification tests

During the experiments, cells were continuously perfused using a peristaltic pump with a standard solution containing (mM): NaCl 127, KCl 3, CaCl_2 _2, MgCl_2 _1.3, HEPES 20, glucose 10; pH was adjusted to 7.4 with NaOH. During the acidification test, the standard solution was replaced with one that had pH adjusted to 5.4 with HCl. All drugs were applied by bath application via the peristaltic pump perfusion. For NMDA receptor activation ("chemical LTP"), cells were perfused for 5 minutes with the extracellular solution lacking Mg^2+ ^and supplemented with 200 μM glycine, 0.5 μM TTX and 200 μM picrotoxin. Time course of solution exchange was estimated by imaging fluorescence changes during wash-in and wash-out of the water soluble fluorescent dye quinacrine. The T_10-90 _of solution exchange was measured as 22 ± 3 s (n = 3).

### 2.5 Data analysis

Images were quantified and processed using Olympus Biosystems AnalySIS software. Background fluorescence was subtracted prior to calculations. The *n *values refer to the number of data points obtained from individual cells in separate culture dishes. Data are presented as mean ± standard error. Plots and figures were constructed using Origin 6.0 software (Microcal) and PowerPoint (Microsoft). For statistical analysis, Student's *t*-test and ANOVA test were used. Curve fitting was performed in Origin 6.0 software using first-order exponential decay fitting and linear fitting procedures.

## Abbreviations

PM: Plasma membrane; TIRF: total internal reflection fluorescence; AMPA: α-amino-3-hydroxyl-5-methyl-4-isoxazole-propionate; NMDA: N-methyl-D-aspartic acid; LTP: long-term potentiation; GluR glutamate receptor; ER: endoplasmic reticulum; EGFP: enhanced green fluorescent protein; KDEL: Lys-Asp-Glu-Leu sequence in the amino acid structure of a protein which keeps it from secreting from the ER; CS: cytosol; cDNA: complementary deoxyribonucleic acid; HEPES: 4-(2-hydroxyethyl)-1-piperazineethanesulfonic acid; TTX: tetrodotoxin; DPSS laser: diode-pumped solid-state laser.

## Authors' contributions

SSK and EP performed the imaging experiments, analyzed the data and participated in writing the manuscript. SKC performed molecular biological experiments and participated in the manuscript revising. AJ and KK designed the cDNA constructs and revised the manuscript. LK conceived of and coordinated the study and drafted the manuscript. All authors read and approved the final manuscript.

## Supplementary Material

Additional file 1 **Figure S1**. Scheme illustrating GluR-A receptor tagged with pHluorin at the N-terminus. Click here for file

Additional file 2 **Figure S2**. The pHluorin-ER fluorescence is detectable in TIRF mode indicating that parts of the ER are close enough to the basal PM to be excited by the evanescent field. Images of a pHluorin-ER transfected neuron obtained in Epifluorescence (Epi), TIRF, or Bright field modes and merged pseudo colored: Epifluorescence (green) and TIRF (red) images (Epi + TIRF). Scale bar: 10 μm. Click here for file
