# Supplementary figures and images for: Dynamic visualization of membrane-inserted fraction of pHluorin-tagged channels using repetitive acidification technique
Source: BMC Neurosci. 2009 Nov 30;10:141. doi: 10.1186/1471-2202-10-141 (PMC2794868; doi:10.1186/1471-2202-10-141)

pHluorin-GluR-A

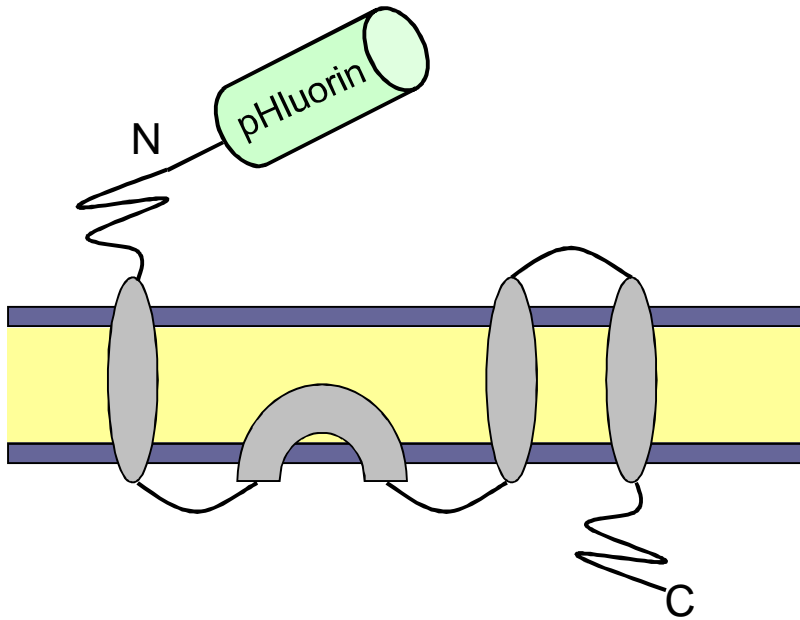

Supplement: Additional file 1 — Figure S1. Scheme illustrating GluR-A receptor tagged with pHluorin at the N-terminus. [file 1471-2202-10-141-S1.pdf]

## pHluorin-ER

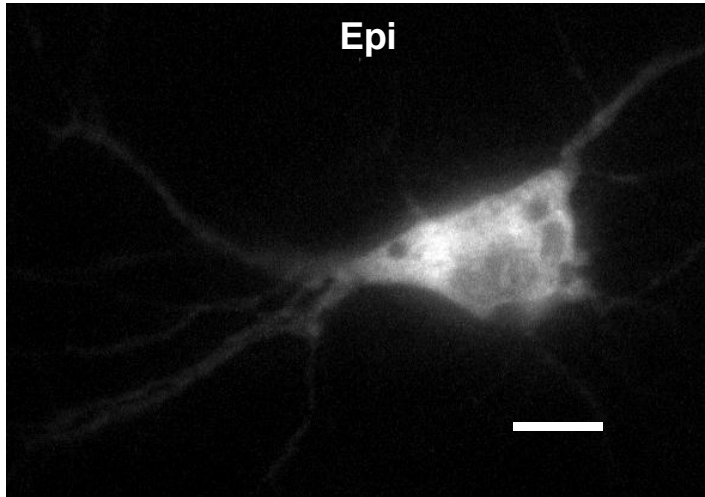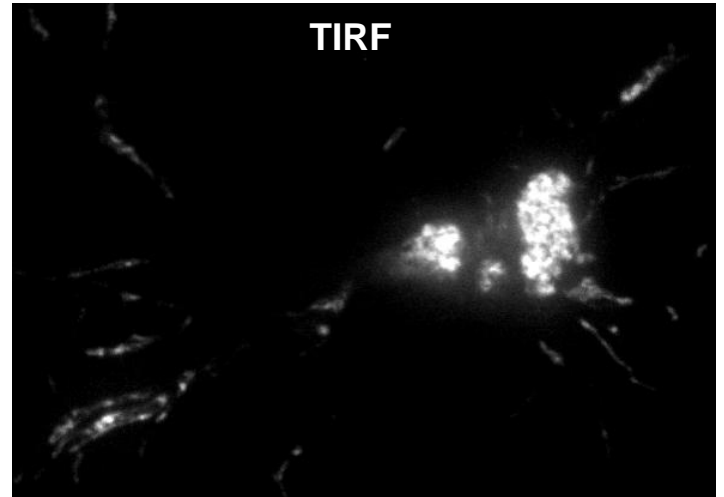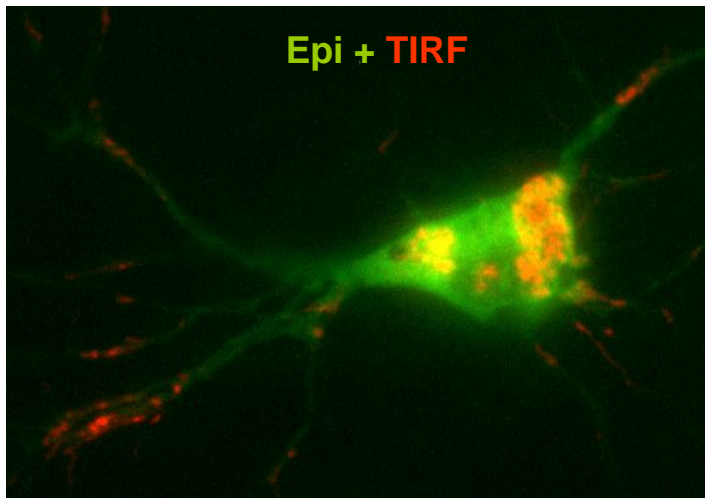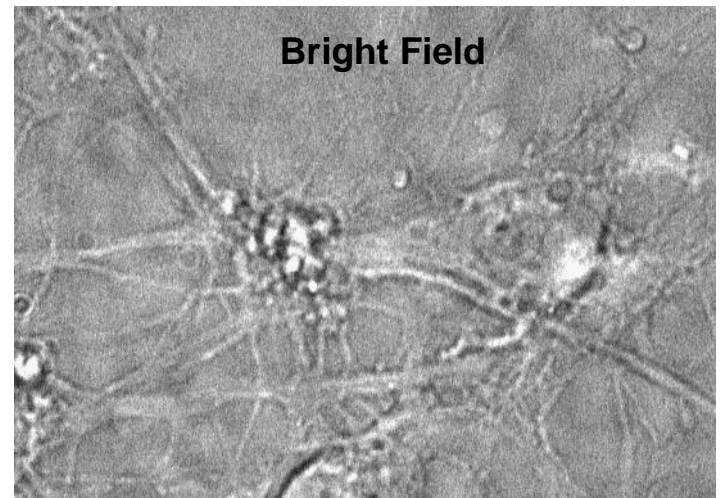

Supplement: Additional file 2 — Figure S2. The pHluorin-ER fluorescence is detectable in TIRF mode indicating that parts of the ER are close enough to the basal PM to be excited by the evanescent field. Images of a pHluorin-ER transfected neuron obtained in Epifluorescence (Epi), TIRF, or Bright field modes and merged pseudo colored: Epifluorescence (green) and TIRF (red) images (Epi + TIRF). Scale bar: 10 μm. [file 1471-2202-10-141-S2.pdf]
